# County-Level Distribution, Relative Abundance and Environmental Associations of *Haemaphysalis longicornis* (Acari: Ixodidae) in the Changbai Mountain Forest Area, Northeastern China

**DOI:** 10.3390/vetsci13101118

**Published:** 2026-10-20

**Authors:** Miao Li, Xiu Xin, Qichao Tang, Xiujing Feng, Hongyu Qin

**Affiliations:** 1 Key Lab of Preventive Veterinary Medicine in Jilin Province, Jilin 132101, China; 2 College of Animal Science and Technology, Jilin Agricultural Science and Technology College, Jilin 132101, China

**Keywords:** *Haemaphysalis longicornis*, tick survey, county-level distribution, spatial statistics, Changbai Mountain, cattle grazing

## Abstract

*Haemaphysalis longicornis* is a three-host tick of cattle and other livestock in East Asia. We surveyed ticks in 19 counties of the Changbai Mountain forest area, northeastern China, from March to October 2023–2025. Ticks were collected by flagging vegetation and from cattle at 103 sampling localities, identified morphologically to species and counted per county; no molecular confirmation of the identifications was performed. County-level counts were analyzed with global and local spatial statistics, a standard deviational ellipse, and rank correlations with eight environmental variables. An exploratory maximum entropy (MaxEnt) model was built on the eight counties in which the species was recorded. Of 1653 ticks belonging to five species, 246 (14.88%) were *H. longicornis*, recorded in 8 of the 19 counties, with the highest relative abundance in Hunchun (81.82%), Tumen (72.84%) and Ji’an (70.63%). Global Moran’s I was 0.0416 (z = 0.84, *p* = 0.402) and local Getis-Ord Gi* identified no significant hot or cold spots, so no statistically significant clustering was detected at the county level; the sampling localities were arranged along an east-northeast to west-southwest axis (SDE azimuth 74.1 degrees). No environmental variable was significantly correlated with county-level relative abundance (all |rho| ≤ 0.33, *p* ≥ 0.16); with 19 counties the test has limited power, so this indicates that no association was detected rather than that none exists. In the exploratory MaxEnt model, four-fold spatial block cross-validation gave an AUC of 0.558 ± 0.113. At this sampling intensity the data support a county-level description of where the species was caught and a preliminary surveillance priority for the three border counties, but not a predictive habitat model.

## Simple Summary

*Haemaphysalis longicornis* is a tick of cattle and other livestock that is native to East Asia and has become established in several other regions of the world. We surveyed ticks at 103 locations in 19 counties of the Changbai Mountain forest area, northeastern China, between 2023 and 2025, collecting them from vegetation and from cattle and identifying them by morphology. Of 1653 ticks belonging to five species, 246 (14.88%) were *H. longicornis*. The species was recorded in 8 of the 19 counties and made up its largest share of the tick catch in the 3 border counties of Hunchun, Tumen and Ji’an (the surveyed counties adjoining the international border). At county level the counts showed no statistically significant clustering, and none of the eight environmental variables we examined was significantly correlated with county-level relative abundance. These results show where the species was caught and how the catch was composed, and they identify the three border counties as the places where surveillance and control are most likely to be worth targeting first, based on unstandardized catch composition. They also show that county-level tick data collected at this intensity cannot resolve the environmental drivers of the species’ distribution.

## 1. Introduction

*Haemaphysalis longicornis* Neumann (Acari: Ixodidae) is a three-host tick that is native to East Asia and has become established, as an introduced species, in several other regions of the world [1,2,3,4,5,6,7,8]. It is a parasite of cattle and other domestic and wild ungulates and can reach high infestation levels on livestock. The tick is also a vector of pathogens of veterinary and public health importance [3], and heavy infestations reduce cattle productivity and cause economic losses to livestock production [1,9]. In China, *H. longicornis* is widely distributed across temperate and subtropical zones but is particularly abundant in the forest–steppe ecotone of northeastern China [10,11]. Recent species distribution models have projected that climate and land-use change may expand its suitable range northward. In Jilin Province, the tick fauna of the eastern mountain region has previously been surveyed [12,13], but the county-level distribution of *H. longicornis* within the Changbai Mountain forest area and its association with the environmental variables available at that scale have not been described, which limits targeted surveillance and control.

Maximum entropy (MaxEnt) modeling is a standard tool for predicting the potential geographic distribution of tick species because it performs well with presence-only data and relatively small sample sizes [14,15]. MaxEnt has been applied to *H. longicornis* in the United States [16,17], Japan [18] and China [19,20,21,22], and a rule-based envelope model has been fitted to the same species in New Zealand [23]; these studies consistently identify temperature, precipitation and land cover as key environmental predictors. Vegetation structure and land use also shape tick habitats by modulating the near-ground microclimate and host availability [24,25]. In China, most existing models for *H. longicornis* have been built at national or provincial scale. County-level surveys in the northeastern forest belt, where livestock production and tick exposure overlap, are still scarce, and the environmental variables that are available at county scale may not resolve the distribution of a tick whose habitat is defined by finer-grained vegetation and host conditions.

To address this gap, we surveyed ticks in 19 counties of the Changbai Mountain forest area over three years, described the species composition of the catch, examined whether the county-level counts of *H. longicornis* are spatially structured, and tested county-level associations between its relative abundance and the environmental variables available at that scale [10,11,14,15,24,25]. Because the survey records are held at county level, the county is the analytical unit throughout, and a maximum entropy model fitted to the county-level records is presented as an exploratory analysis in the Supplementary Materials. The results identify where *H. longicornis* was caught and which counties should be prioritized for surveillance in cattle grazing systems.

## 2. Materials and Methods

### 2.1. Study Area

This study encompassed 19 counties and county-level cities in eastern Jilin Province, northeastern China (41°08′–44°40′ N, 125°16′–131°18′ E), including the Changbai Mountain Nature Reserve and its surrounding forest, agricultural, and pastoral landscapes. The region has a temperate continental monsoon climate with mean annual temperatures ranging from 2 to 6 °C and annual precipitation from 600 to 1000 mm. Elevation ranges from approximately 100 m in the western plains to over 2600 m at the summit of Changbai Mountain. The dominant vegetation types include Korean pine broad-leaved mixed forest, secondary deciduous forest, shrubland, and riparian meadow. Cattle are grazed extensively in the forest understory and along the forest margins from spring through autumn, where they are the principal hosts for the adult ticks.

### 2.2. Tick Collection and Identification

Ticks were collected at 103 sampling localities in 19 counties of eastern Jilin Province (Table 1; Supplementary Table S1) between March and October of 2023–2025. Within each county, five to six localities were selected to represent the dominant land-cover types (forest, shrubland, meadow and grazing land). At each locality, questing ticks were collected by flagging vegetation and leaf litter with a 90 × 60 cm white cotton cloth for at least two person-hours per visit [26], and feeding ticks were removed from naturally infested cattle with fine forceps. All specimens were examined under a stereomicroscope and their species were identified using standard morphological keys for the adult Ixodidae of China [27]; the material examined consisted of adult ticks. Identification of adult ixodids with these keys is an established approach in regional tick surveys [12], and no molecular confirmation was performed. Immature stages (larvae and nymphs), for which morphological identification is not reliable, were not part of the dataset, so no species assignment in this study depends on immature specimens. The numbers of ticks collected by the two methods were not recorded separately, and the counts presented here are county-level totals of all specimens obtained by either method. Species records are held at county level; the localities are sampling sites, not occurrence records for individual species.

### 2.3. Environmental Variables

Data for nineteen bioclimatic variables, along with elevation, were obtained from WorldClim 2.1 at a 2.5-arc-minute resolution (≈5 km). Land cover was obtained from the ESRI Sentinel-2 10 m annual land-cover product (2023) and aggregated to the same 2.5-arc-minute grid by majority rule. The normalized difference vegetation index (NDVI) was derived from the MODIS MOD13A1 product (500 m, 2023), using the maximum-value composite of the growing season (April to October) to represent peak-season vegetation greenness. All layers were clipped to the union of the 19 surveyed counties, which defines the accessible area (5641 grid cells with complete values for all eight environmental variables), and reprojected to WGS 1984 UTM Zone 52N. The accessible area and the background used in the exploratory model differ by the eight presence cells of the recorded counties (5633 background cells); both values are used consistently throughout the manuscript. To reduce multicollinearity, R (version 4.6.1; R Foundation for Statistical Computing, Vienna, Austria) with the usdm package (version 2.1.7) was employed to conduct VIF (Variance Inflation Factor) and Pearson correlation coefficient tests on the variables. The threshold criteria for the tests were set as VIF values less than 10 and absolute values of Pearson correlation coefficients less than 0.8 between variables. The eight variables used in the analyses are shown in Table 2.

### 2.4. Spatial Analysis

During sampling, the geographic coordinates of each collection site were recorded with a handheld GPS receiver (Table 1). In this manuscript, “tick occurrence” denotes the records of the five tick species identified at the sampling localities; the spatial statistics use the county-level counts and the environmental association analysis uses the county-level relative abundance of *H. longicornis*, the target species of the study. Global spatial autocorrelation of *H. longicornis* counts across the 19 surveyed counties was assessed with Global Moran’s I in ArcGIS 10.8 (Esri, Redlands, CA, USA). A spatial weights matrix based on the four nearest neighbors with row standardization was generated with the Generate Spatial Weights Matrix tool, and significance was evaluated from the z-score of the statistic. Local cluster analysis was performed with the Hot Spot Analysis (Getis-Ord Gi*) tool using the same weights matrix [28], and counties were classified as significant hot or cold spots at the 90%, 95%, and 99% confidence levels. Unsurveyed counties were excluded from the analysis, so no unsurveyed area was interpreted as a true low-occurrence area. A one-standard-deviation standard deviational ellipse (SDE) fitted to the 103 georeferenced sampling localities was used to describe the directional arrangement of the sampling sites.

### 2.5. County-Level Environmental Association Analysis and Exploratory Habitat Modeling

For each of the 19 surveyed counties, the mean value of each of the eight environmental variables was calculated from the 2.5-arc-minute grid. For land cover, whose classes are nominal, the county value is the mean of the majority-rule class codes, and the rank correlation involving it is therefore reported as a descriptive summary rather than as a formal test. Because the survey records are held at county level (Section 2.2), the county is the unit of analysis. Spearman rank correlations between the county-level relative abundance of *H. longicornis* and each environmental variable were computed across the 19 counties; with this number of units the *p*-values are descriptive and were not corrected for multiple comparisons. To describe how much each variable actually varied across the surveyed belt, the coefficient of variation of its 19 county means was calculated as the standard deviation divided by the mean. An exploratory maximum entropy model (MaxEnt 3.4.4; R Foundation for Statistical Computing, Vienna, Austria) was also fitted, with each of the eight counties in which *H. longicornis* was recorded contributing a single presence cell at its reference coordinate (Table 1), so that the 5641 cells of the accessible area yielded 5633 background cells, and with occurrence records checked for minimum separation [29,30]. The background was defined by the accessible area [31], and feature classes and the regularization multiplier were tuned over 20 combinations [32,33] (the codes in Supplementary Table S3 denote the features tested: L = linear, Q = quadratic, H = hinge, and P = product). Model performance was evaluated by four-fold spatial block cross-validation and leave-one-out cross-validation [34]. The settings, tuning table and outputs of this exploratory model are provided in the Supplementary Materials (Table S3; Figures S1–S4); because only eight presence units were available, no inference in the main text rests on it. Response curves are reported for descriptive purposes only [35].

## 3. Results

### 3.1. Tick Species Composition and Relative Abundance

**Figure 1:**
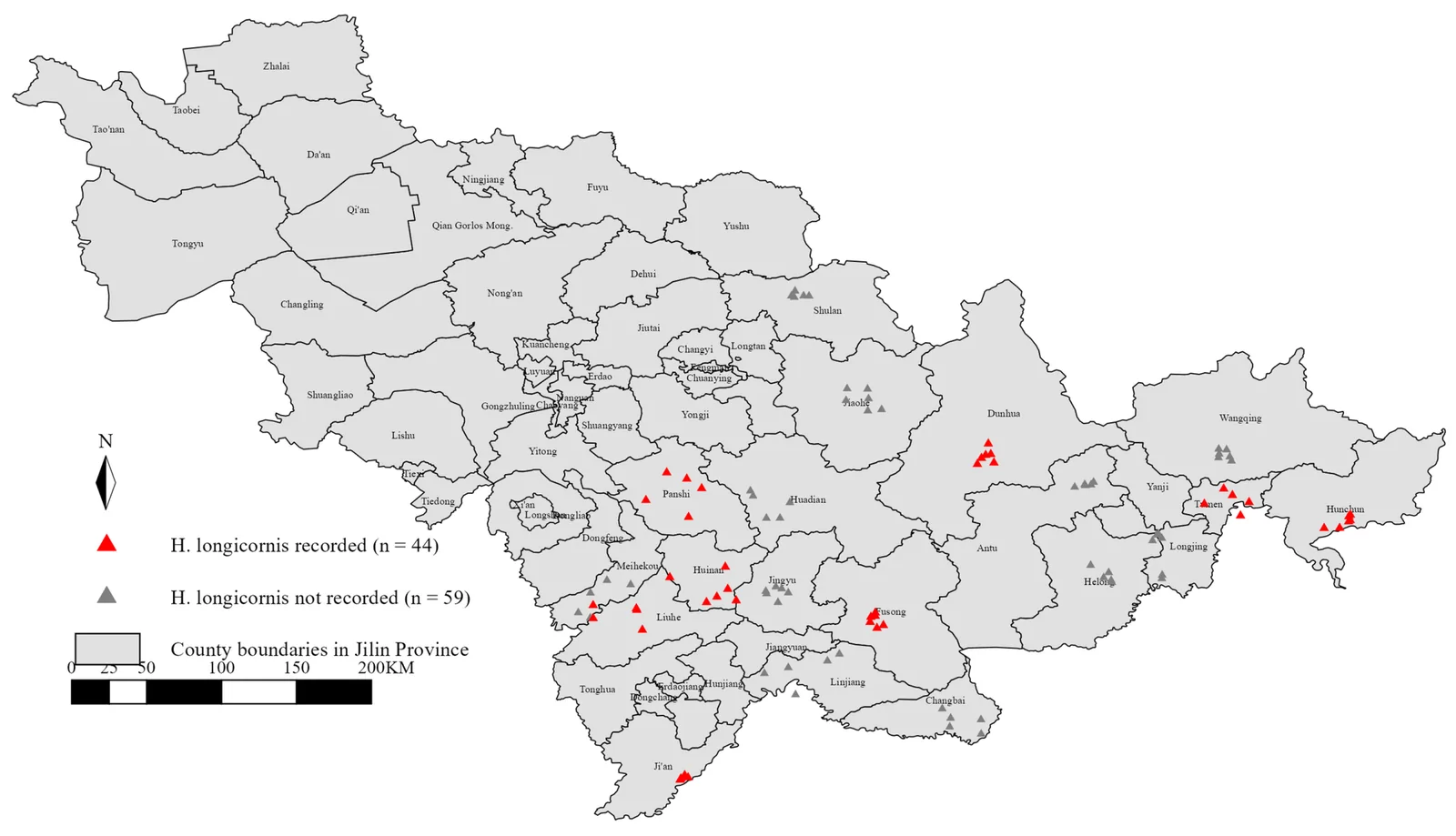
Sampling localities for tick collection in the Changbai Mountain forest area, northeastern China, 2023–2025. Note: red triangles denote localities in counties where *H. longicornis* was recorded (n = 44) and dark grey triangles those in counties where it was not (n = 59); the symbols describe the county of each locality and not the species caught at that locality.

A total of 1653 ticks comprising three genera and five species were collected. *Ixodes persulcatus* was the most abundant (517 specimens, 31.28%), followed by *Haemaphysalis concinna* (432, 26.13%), *Dermacentor nuttalli* (288, 17.42%), *H. longicornis* (246, 14.88%), and *D. silvarum* (170, 10.28%). Specifically, *H. longicornis* was present in 8 of the 19 sampled counties: Hunchun (81/99, 81.82%), Tumen (59/81, 72.84%), Ji’an (89/126, 70.63%), Panshi (11/72, 15.28%), Dunhua (2/92, 2.17%), Huinan (2/74, 2.70%), Fusong (1/97, 1.03%), and Liuhe (1/93, 1.08%). Counties in which only one or two *H. longicornis* specimens were recorded (Dunhua, Huinan, Fusong and Liuhe) rest on very small counts, and their abundance estimates are correspondingly uncertain. The county-level counts of *H. longicornis* and of all ticks are summarized in Table 1, and the geographic distribution of the collection localities is shown in Figure 1.

Based on field observations during the survey period, adult *H. longicornis* were active from March through June, with questing activity apparently peaking from mid-March to early May. In comparison, *Dermacentor* spp. emerged immediately after snowmelt and declined by late April, while the activity of *I. persulcatus* and the other *Haemaphysalis* species (mainly *H. concinna*) peaked from April to June. These phenological patterns come from field observations, not standardized monthly counts.

### 3.2. Spatial Distribution Pattern

**Figure 2:**
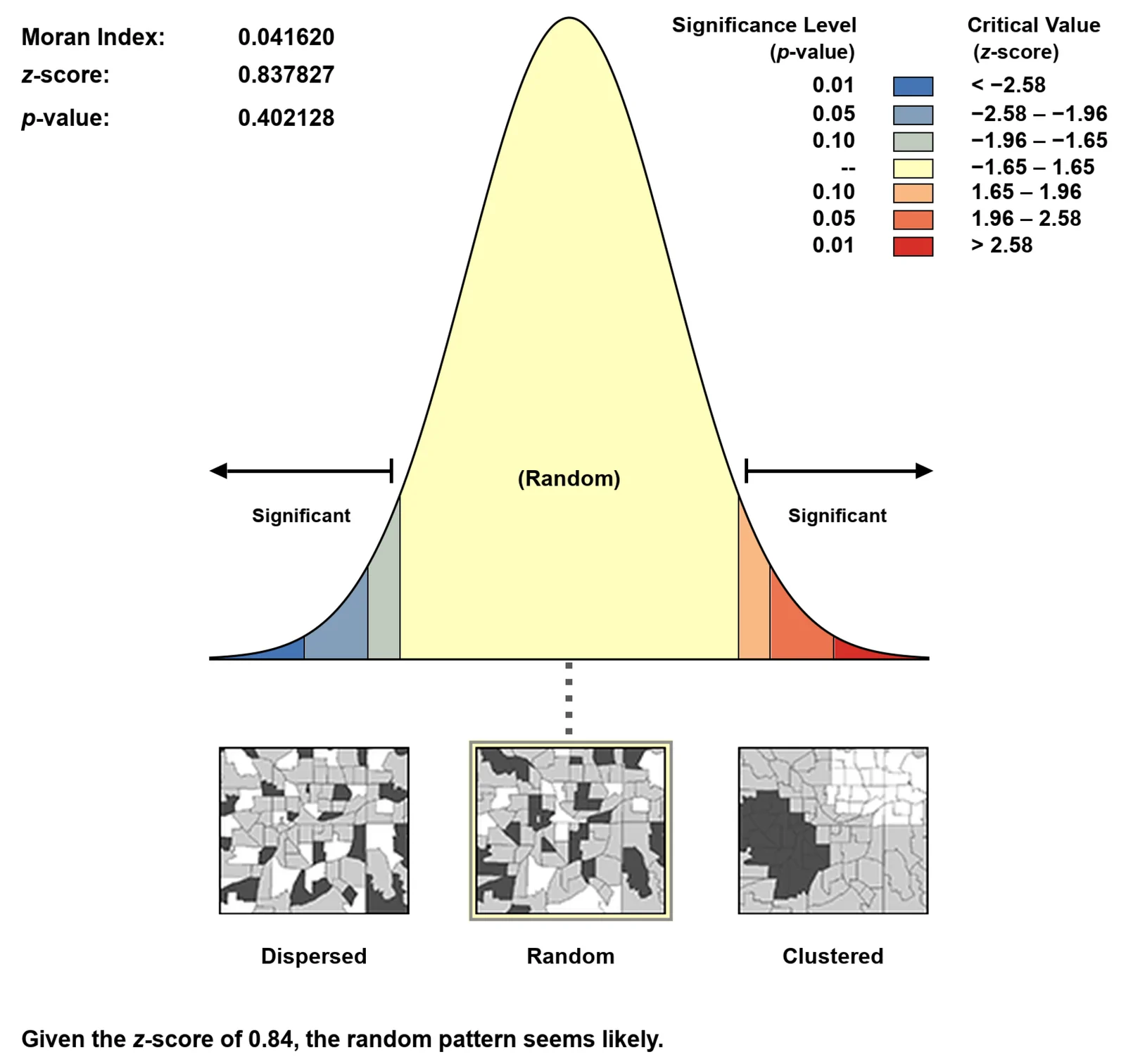
Null distribution of Global Moran’s I for the 19 surveyed counties (ArcGIS Desktop 10.8, Spatial Autocorrelation tool). Note: the shaded tails mark the significant ranges; “--“ marks the central band (−1.65 < z < 1.65), the non-significant range, which carries no single *p*-value; the three lower panels show dispersed, random and clustered patterns.

Across the 19 surveyed counties, the global spatial pattern of *H. longicornis* counts did not differ from randomness. Global Moran’s I computed with a four-nearest-neighbor row-standardized weights matrix was 0.0416 (z = 0.84, *p* = 0.402), against an expected value of −0.0556 under the null hypothesis of complete spatial randomness (Figure 2). No statistically significant global spatial autocorrelation was detected at the county scale. With only 19 analysis units, 11 of which carry a zero count, the test has limited power, so this result indicates that clustering was not detected rather than that it is absent.

**Figure 3:**
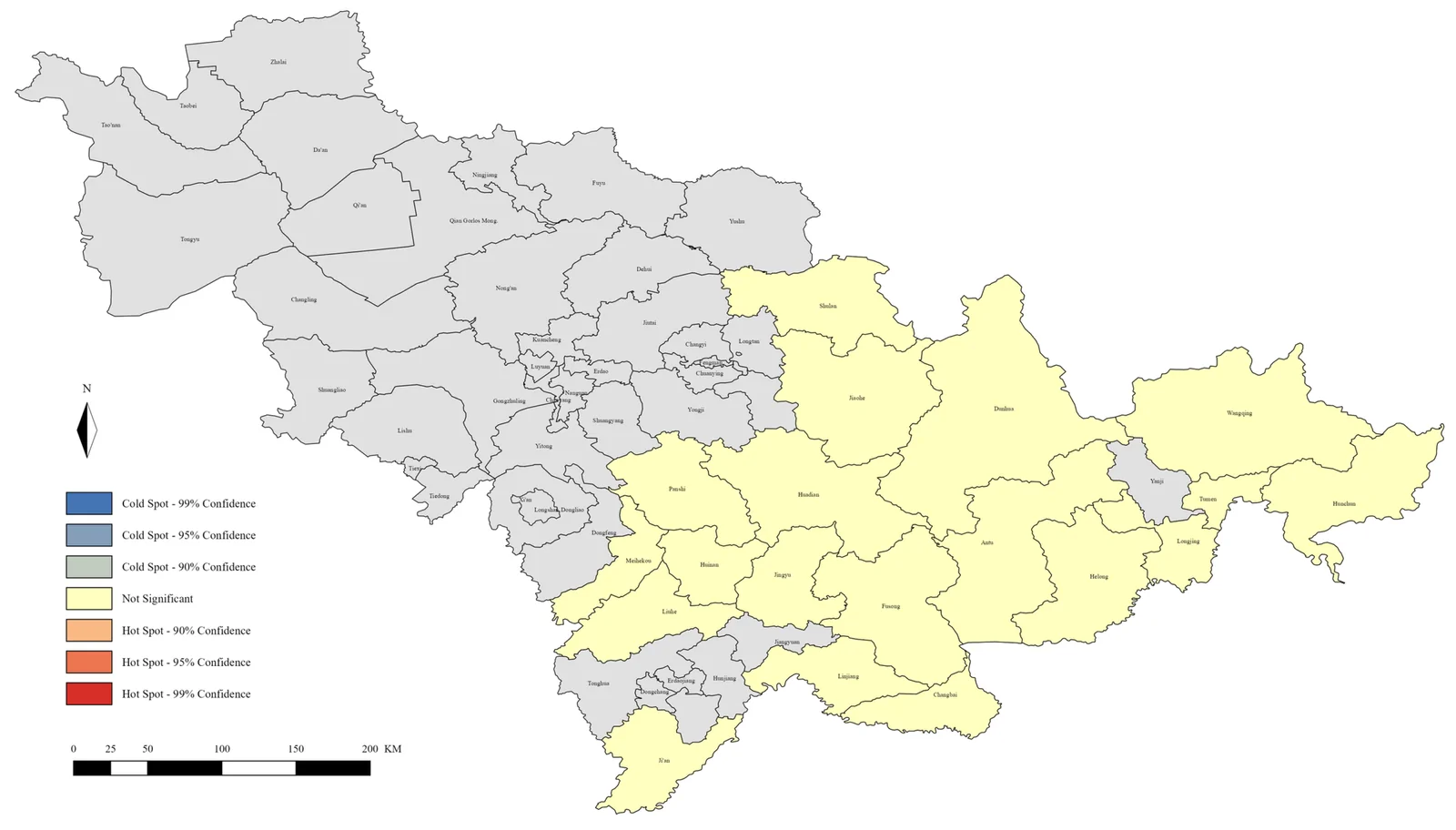
Local hotspot analysis (Getis-Ord Gi*) of *H. longicornis* counts across the 19 surveyed counties. Note: unsurveyed counties are shown in grey and were excluded from the analysis.

Local cluster analysis with the Getis-Ord Gi* statistic identified no statistically significant hot spots or cold spots among the 19 surveyed counties (Figure 3); all counties were classified as “Not Significant” at the 90%, 95%, and 99% confidence levels. The highest absolute Gi* z-score was 1.36 (Hunchun and Tumen), well below the 1.65 threshold for the 90% confidence level. These results indicate that the between-county variation in *H. longicornis* relative abundance (0–81.82% of total ticks), although large in relative terms, was not accompanied by statistically detectable local clustering at this number of analysis units.

**Figure 4:**
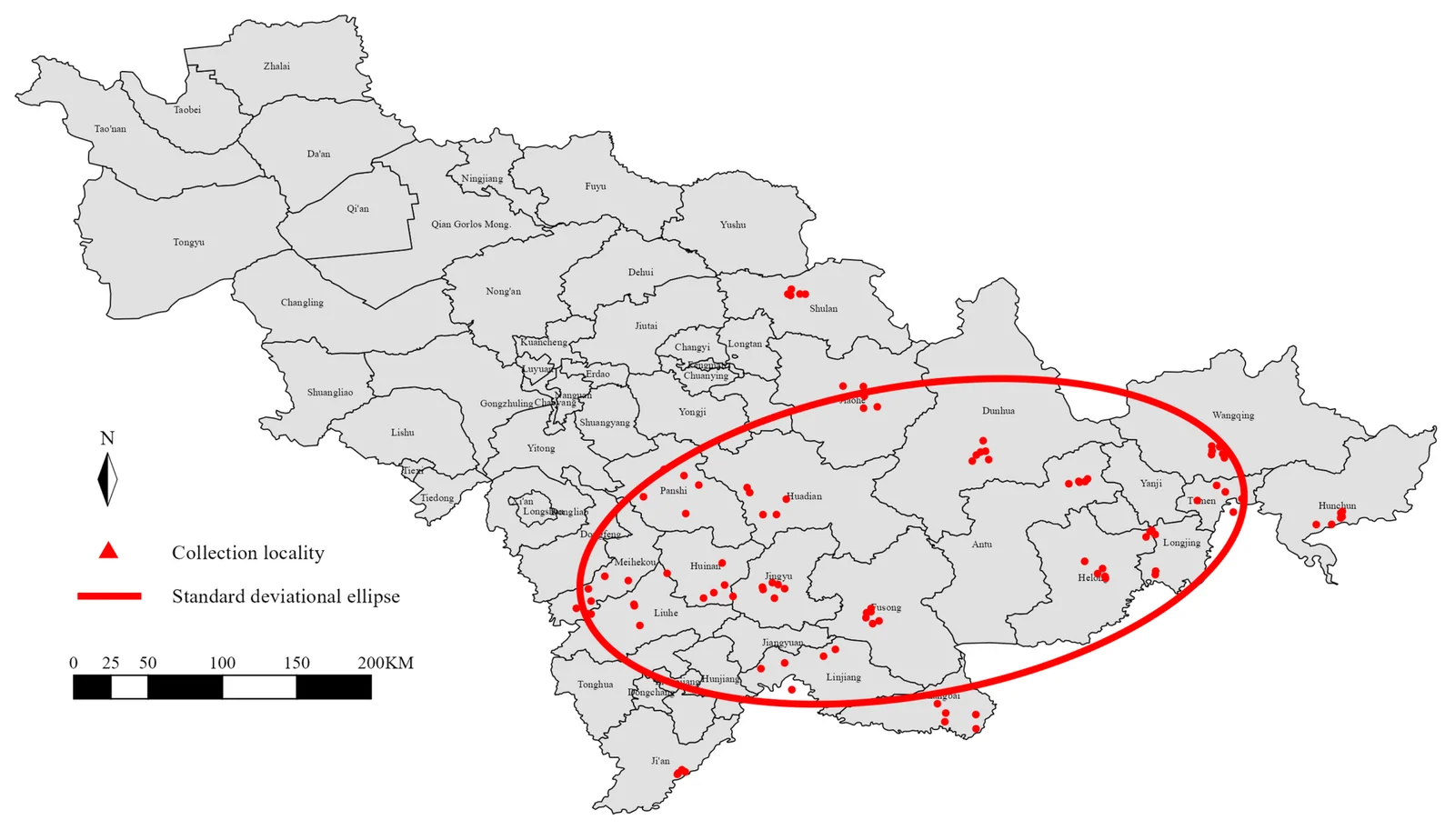
Standard deviational ellipse (SDE) of the 103 tick collection localities in the Changbai Mountain forest area. Note: red markers indicate sampling sites and the red ellipse is the one-standard-deviation ellipse.

The one-standard-deviation standard deviational ellipse fitted to the 103 collection localities was elongated along an east-northeast to west-southwest axis (azimuth 74.1° from north). The major axis measured 250.9 km and the minor axis 151.9 km, and the ellipse was centered at 127.734° E, 42.707° N (Figure 4). The ellipse enclosed 31% of the sampling localities, close to the 39% expected for a one-standard-deviation ellipse under a bivariate normal distribution; its orientation reflects the ENE–WSW arrangement of the surveyed area along the Changbai Mountain foothills.

### 3.3. Environmental Variable Selection

After collinearity screening, eight environmental variables were retained: mean diurnal temperature range (bio_2), maximum temperature of the warmest month (bio_5), minimum temperature of the coldest month (bio_6), precipitation of the driest month (bio_14), precipitation seasonality (bio_15), elevation, land cover, and NDVI (Table 2).

### 3.4. County-Level Environmental Associations

Across the 19 counties, no environmental variable was significantly correlated with the relative abundance of *H. longicornis* (Table 3). The strongest correlations were negative for elevation (rho = −0.33, *p* = 0.16) and mean diurnal temperature range (rho = −0.28, *p* = 0.24) and positive for minimum temperature of the coldest month (rho = 0.25, *p* = 0.29). The three counties with the highest relative abundance did not share an extreme value of any single variable. With 19 units, the test has limited power: at this sample size a rank correlation would generally be detected only if it were strong, so these results indicate that no association was detected rather than that none exists.

### 3.5. Exploratory Habitat-Suitability Modeling

Under four-fold spatial block cross-validation, the exploratory MaxEnt model fitted to the eight county-level occurrence records achieved a mean AUC of 0.558 ± 0.113 (range across the 20 candidate parameterizations 0.375–0.609), and leave-one-out cross-validation, in which each fold holds a single presence unit, gave a mean AUC of 0.648 across the eight folds; the block estimate is the more reliable of the two, because a fold containing a single presence unit constrains the possible AUC values; seven of the 20 parameterizations returned an AUC of 0.500. Detailed results, including the tuning table, response curves and the suitability surface, are provided in the Supplementary Materials (Table S3; Figures S1–S4). We treat the modeled surface as exploratory and do not draw surveillance or management conclusions from it.

## 4. Discussion

This study describes the tick fauna of 19 counties in the Changbai Mountain forest area and the county-level distribution of *H. longicornis* over three survey years. *Haemaphysalis longicornis* accounted for 14.88% of the 1653 ticks collected and reached its highest relative abundance in Hunchun (81.82%), Tumen (72.84%) and Ji’an (70.63%). At county level, neither Global Moran’s I (I = 0.0416, *p* = 0.402) nor local Gi* identified statistically significant clustering, so the concentration in three counties is a difference in relative abundance and not statistically significant clustering. The habitat requirements described for *H. longicornis* are consistent with its presence in these counties: the species is associated with moderate to high rainfall, a well-developed understory, and abundant large mammalian hosts [11,36]. Counties north and west of the surveyed belt may offer less shrub-forest edge habitat and fewer grazing hosts [37], but this remains a hypothesis because host density and grazing intensity were not measured and no county outside the surveyed belt was examined. Because sampling followed the mountain foothills where cattle are grazed in the forest understory, it covered the putative range of the species and was not designed as an unbiased sample of the province [38,39]; environmental surveys around *H. longicornis*-positive sites in North America and tick-exposure studies in northeastern China document the species in comparable habitat and host contexts [38,39].

None of the eight environmental variables was significantly correlated with the county-level relative abundance of *H. longicornis* (Table 3), and the county means of most variables varied very little across the surveyed belt (coefficient of variation 0.015 for NDVI, 0.035 for precipitation seasonality, 0.047 for maximum temperature of the warmest month and 0.049 for mean diurnal temperature range). This is consistent with the scale dependence of environmental associations reported for this species: studies that detect temperature-, precipitation- or land-cover-driven associations for *H. longicornis* [19,20,21,22] and for other ixodid ticks [40,41,42,43] model distributions at national or continental scale, where those variables carry a gradient that a single humid forest belt does not. Comparable analyses at the same scale have reached the same conclusion: in the southeastern United States, habitat and vegetation variables were insufficient to predict the abundance of three tick species and no spatial structure was evident [44], whereas a fine-scale survey of *H. longicornis* in the environmentally heterogeneous mountains of Shandong, China, detected clear environmental correlates and achieved good discrimination [45]. Within the 19 surveyed counties, the climate variables that dominate such models are therefore close to invariant, and three further features of the dataset limit what this design can resolve. First, the county is the unit of analysis and only 19 units are available, so power is low and county means smooth over the vegetation and microclimate gradients that operate at the scale of a tick’s habitat. Second, the relative abundance recorded for a county depends on sampling effort, host availability and grazing management, none of which was standardized or measured. Third, only three counties carried a relative abundance above 70% while eleven recorded none at all, so the response is close to zero-inflated and rank-based tests have little power to detect an association even where one exists. The eleven counties with no records are treated as non-detections rather than as confirmed absences, because sampling effort was not standardized and the localities within a county were not a random sample of it. The three counties with the highest relative abundance did not share an extreme value of any single variable, which is more consistent with local host and management conditions than with county-level climate. Where tick abundance has been resolved at finer spatial scales, it is jointly determined by environmental, host and land-use factors rather than by climate alone [46], and the predictive value of environmental variables differs between tick life stages [47]. The practical implication is that coarse-resolution climatic layers are unlikely to guide surveillance for this species within a single climatic belt, and that finer-scale host density, microclimate and grazing management should be measured if the drivers of local abundance are to be identified.

The biology of *H. longicornis* remains relevant to where the species was caught even though the county-level data do not resolve environmental drivers. The tick is susceptible to desiccation and depends on sustained near-ground humidity when off the host [48,49]; saturation deficit suppresses questing activity in ixodid ticks [50], and seasonal studies in Korea and Japan emphasize humidity-related drivers of tick abundance [18,51]. The cool, humid summers of the Changbai Mountain area are expected to keep saturation deficits within a tolerable range for much of the active season, which would be compatible with the species being present throughout the eastern forest belt in this survey; this remains a plausible but untested interpretation, since no humidity was measured at the collection sites. Testing this interpretation would require microclimate measurements at the scale of individual sites, which were not made here.

The concentration of *H. longicornis* in three border counties has practical implications for livestock management. Cattle grazed in the forest understory or along shrub–forest edges are exposed to questing ticks, and Hunchun, Tumen and Ji’an are among the counties where this grazing system is practiced. Zheng et al. found shrubs to be the most suitable biotope for *H. longicornis* among four habitat types surveyed in northern China [36], and environmental predictors have been used to target surveillance of this species in the United States [52]. The border counties lie within the Tumen River basin, where tick-borne viruses have been detected in both ticks and livestock [53]; targeting surveillance at this grazing system is therefore warranted. On the basis of the observed pattern, tick control and pasture rotation focused on the border counties may be more efficient to target than area-wide treatment, and life-stage-targeted acaricide application in spring, when adults are active, is worth evaluating [54]. Because the county ranking depends on sampling effort that was not standardized, it is a first indication of priority rather than a quantitative risk map.

Several limitations should be acknowledged. First, the exploratory model rests on only eight county-level occurrence records and discriminates poorly (block cross-validation AUC 0.558; 7 of the 20 parameterizations returned AUC 0.500); it is presented in the Supplementary Materials and no conclusion here depends on it [55,56,57]. Second, sampling was concentrated along accessible roads and forest-grazing areas, which can bias abundance estimates and any presence-based model [58,59,60]. Third, species records are held at county level: the 103 sampling localities carry coordinates but no per-locality species list, the numbers of ticks obtained by flagging vegetation and from cattle were not recorded separately, and per-visit sampling effort was neither standardized nor retained. All analyses therefore use county-level counts and site-level analysis is not possible; the absence of raw, locality-level records is a recognized obstacle in tick research and has prompted calls for standardized recording and data sharing [61]. Fourth, relative abundance depends on the number and timing of visits, the number of collectors and the number of cattle examined, none of which were recorded, so the ranking of counties by relative abundance should be read with that caveat. Fifth, host-density, grazing-intensity and microclimate variables were not measured, although host distribution influences tick abundance independently of climate and vegetation. Only adult ticks were recorded, so the survey cannot be used to infer the total annual parasitic burden on cattle. Species assignments rest on morphological identification of adult specimens; molecular verification was not performed, so misidentification of damaged or atypical specimens cannot be excluded. Finally, the environmental layers available at 2.5-arc-minute resolution are coarse relative to the scale at which ticks experience habitat, and the exploratory model describes the conditions associated with the recorded counties only; it does not project future scenarios [62].

## 5. Conclusions

This survey documented the tick fauna of 19 counties in the Changbai Mountain forest area and the county-level distribution of *H. longicornis* over three years. *Haemaphysalis longicornis* made up 14.88% of the 1653 ticks collected and reached its highest relative abundance in the border counties Hunchun, Tumen and Ji’an; this concentration is a difference in relative abundance and not statistically significant clustering, since neither Global Moran’s I nor local Gi* indicated spatial structure, and none of the eight environmental variables examined was significantly correlated with relative abundance. On that basis the border counties are a first priority for tick surveillance in cattle grazing systems, and the county-level records available for this species are too coarse and too few to support a predictive habitat model. Because the eight climatic and land-cover variables examined carried almost no variation within this single humid forest belt, coarse-resolution layers of this kind are unlikely to resolve the drivers of local abundance; resolving them will require locality-level records collected with standardized effort, together with host-density and microclimate measurements. Pathogen screening, and the resulting veterinary risk assessment, were outside the scope of this survey.

## Figures and Tables

**Table 1:** County-level reference coordinates and tick counts for the 19 surveyed counties in eastern Jilin Province, China, 2023–2025. Note: counts are county-level totals for the entire survey period.

County/City	Longitude (° E)	Latitude (° N)	*H. longicornis*	Total Ticks	Proportion (%)
Antu	128.881	43.118	0	79	0
Dunhua	128.208	43.324	2	92	2.17
Helong	129.059	42.463	0	94	0
Hunchun	130.658	42.875	81	99	81.82
Longjing	129.396	42.762	0	70	0
Tumen	129.986	43.005	59	81	72.84
Wangqing	129.834	43.360	0	102	0
Changbai	128.177	41.437	0	90	0
Fusong	127.432	42.229	1	97	1.03
Huadian	126.637	43.048	0	73	0
Huinan	126.322	42.329	2	74	2.70
Ji’an	126.150	41.136	89	126	70.63
Jiaohe	127.412	43.624	0	89	0
Jingyu	126.805	42.328	0	83	0
Linjiang	126.713	41.848	0	97	0
Liuhe	125.557	42.308	1	93	1.08
Meihekou	125.650	42.477	0	78	0
Panshi	126.054	43.206	11	72	15.28
Shulan	126.920	44.434	0	64	0

**Table 2:** Environmental variables retained after collinearity screening and used in the county-level analyses.

Variable	Description	Unit	Source	Resolution
bio_2	Mean diurnal temperature range	°C	WorldClim 2.1	2.5 arc-min
bio_5	Max temperature of warmest month	°C	WorldClim 2.1	2.5 arc-min
bio_6	Min temperature of coldest month	°C	WorldClim 2.1	2.5 arc-min
bio_14	Precipitation of driest month	mm	WorldClim 2.1	2.5 arc-min
bio_15	Precipitation seasonality (coefficient of variation)	%	WorldClim 2.1	2.5 arc-min
elev	Elevation	m	WorldClim 2.1	2.5 arc-min
landcover	Land cover (9 classes)	category	ESRI Sentinel-2 (2023)	10 m, aggregated
NDVI	Growing-season maximum normalized difference vegetation index	0–1	MODIS MOD13A1 (2023)	500 m, resampled

**Table 3:** Spearman rank correlations between the county-level relative abundance of *Haemaphysalis longicornis* and the eight environmental variables (n = 19).

Variable	Spearman Rho	*p* Value
Elevation	−0.33	0.16
Mean diurnal temperature range (bio_2)	−0.28	0.24
Minimum temperature of coldest month (bio_6)	0.25	0.29
Max temperature of warmest month (bio_5)	0.22	0.36
Land cover	0.23	0.34
NDVI	0.11	0.65
Precipitation seasonality (bio_15)	−0.05	0.84
Precipitation of driest month (bio_14)	0.03	0.90

## Data Availability

The original contributions presented in this study are included in the article/supplementary material. Further inquiries can be directed to the corresponding author.

## Supplementary Materials

The following supporting information can be downloaded at: https://www.mdpi.com/article/10.3390/vetsci13101118/s1, Table S1 (Supplementary_Table_S1.xlsx): geographic coordinates of the 103 sampling localities. Table S2 (Supplementary_Table_S2.xlsx): county-level occurrence records and environmental means for the 19 surveyed counties; relative abundance is the county count of *H. longicornis* divided by the county total tick count. Table S3 (Supplementary_Table_S3.xlsx): the tuning table for the 20 candidate parameterizations of the exploratory model, the spatial block assignment, and the full model settings. Figure S1: response curves of the eight environmental variables in the exploratory model. The curves are drawn on the logistic output scale, the shaded bands are the standard deviation across 10 bootstrap replicates, and the land-cover panel uses the ESRI Sentinel-2 class codes listed in Table 2 (the coding is not contiguous, which is why the axis runs from 0 to 12 for nine classes). These curves are descriptive only and are not evidence that any variable drives the distribution. Figure S2: exploratory habitat-suitability surface. Values are shown only within the 19 surveyed counties; unsurveyed counties are left unfilled and were assigned no suitability value. The surface has no predictive validity: with eight presence units the model discriminates at chance level (block cross-validation AUC 0.558; seven of the 20 candidate parameterizations returned 0.500), and the mismatch with the observed county counts shown in Figure S3 is expected rather than anomalous. Figure S3: county-level relative abundance of *H. longicornis* in the 19 surveyed counties (observed values). Counties are shaded in five classes, and the three border counties with the highest values are labeled with their exact percentages. Figure S4: receiver operating characteristic (ROC) curves of the exploratory model under four-fold spatial block cross-validation. Each panel-listed fold is plotted separately and the heavy black line is the mean curve; the mean area under the curve is 0.558 (fold values: 0.526, 0.568, 0.433 and 0.705). With two occurrence units held out per fold the curves are coarse and the AUC is unstable, which is the quantitative statement of the weak discrimination referred to in Section 3.5 and Section 4.
