# Supplementary material for: County-Level Distribution, Relative Abundance and Environmental Associations of Haemaphysalis longicornis (Acari: Ixodidae) in the Changbai Mountain Forest Area, Northeastern China
Source: Vet Sci. 2026 Oct 20;13(10):1118. doi: 10.3390/vetsci13101118 (PMC13709795; doi:10.3390/vetsci13101118)
Supplement: Supplementary file 1 [file vetsci-13-01118-s001.zip › Figure S1.pdf]

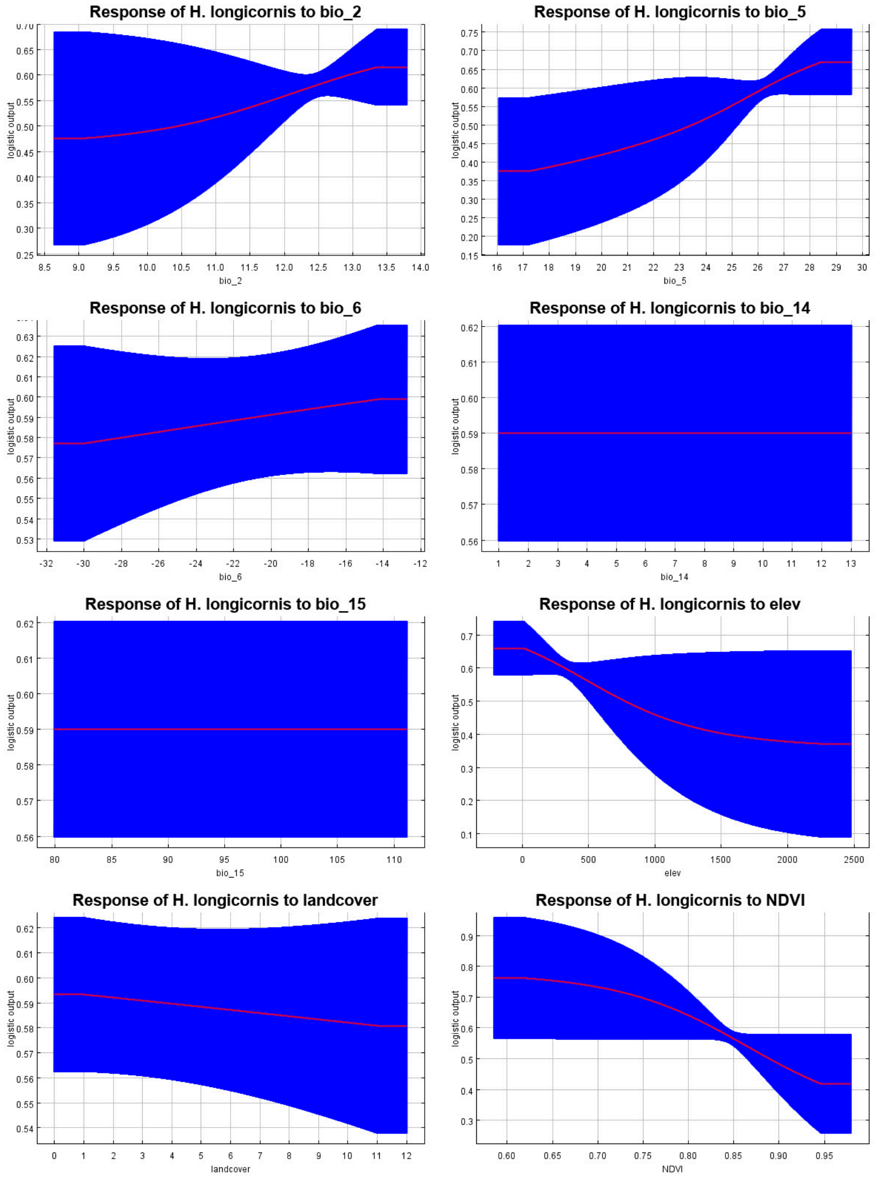

Figure S1. Response curves of the eight environmental variables in the exploratory model. The curves are drawn on the logistic output scale, the shaded bands are the standard deviation across 10 bootstrap replicates, and the land-cover panel uses the ESRI Sentinel-2 class codes listed in Table 2 (the coding is not contiguous, which is why the axis runs from 0 to 12 for nine classes). These curves are descriptive only and are not evidence that any variable drives the distribution.
