# Supplementary material for: County-Level Distribution, Relative Abundance and Environmental Associations of Haemaphysalis longicornis (Acari: Ixodidae) in the Changbai Mountain Forest Area, Northeastern China
Source: Vet Sci. 2026 Oct 20;13(10):1118. doi: 10.3390/vetsci13101118 (PMC13709795; doi:10.3390/vetsci13101118)
Supplement: Supplementary file 1 [file vetsci-13-01118-s001.zip › Figure S2.pdf]

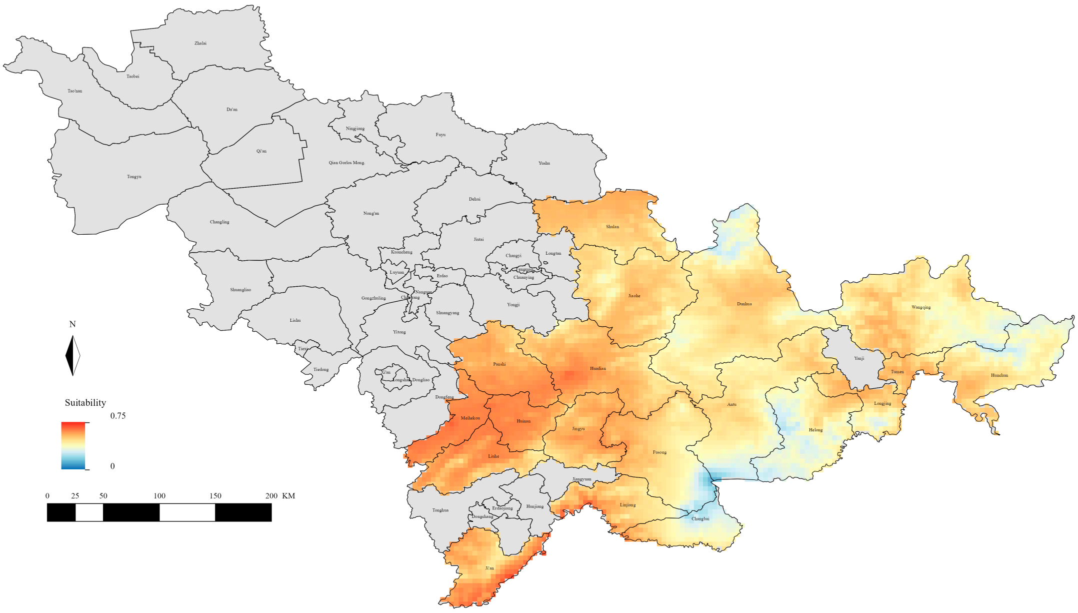

Figure S2. Exploratory habitat-suitability surface. Values are shown only within the 19 surveyed counties; unsurveyed counties are left unfilled and were assigned no suitability value. The surface has no predictive validity: with eight presence units the model discriminates at chance level (block cross-validation AUC 0.558; seven of the 20 candidate parameterizations returned 0.500), and the mismatch with the observed county counts shown in Figure S3 is expected rather than anomalous.
