# Supplementary material for: County-Level Distribution, Relative Abundance and Environmental Associations of Haemaphysalis longicornis (Acari: Ixodidae) in the Changbai Mountain Forest Area, Northeastern China
Source: Vet Sci. 2026 Oct 20;13(10):1118. doi: 10.3390/vetsci13101118 (PMC13709795; doi:10.3390/vetsci13101118)
Supplement: Supplementary file 1 [file vetsci-13-01118-s001.zip › Figure S3.pdf]

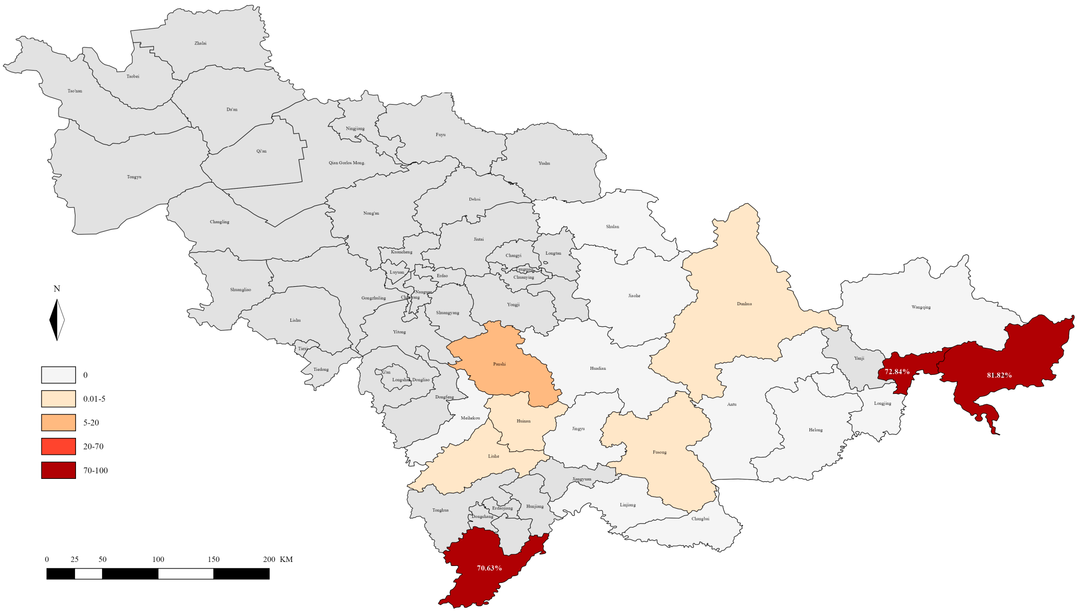

Figure S3. County-level relative abundance of H. longicornis in the 19 surveyed counties (observed values). Counties are shaded in five classes, and the three border counties with the highest values are labeled with their exact percentages.
