# Supplementary material for: County-Level Distribution, Relative Abundance and Environmental Associations of Haemaphysalis longicornis (Acari: Ixodidae) in the Changbai Mountain Forest Area, Northeastern China
Source: Vet Sci. 2026 Oct 20;13(10):1118. doi: 10.3390/vetsci13101118 (PMC13709795; doi:10.3390/vetsci13101118)
Supplement: Supplementary file 1 [file vetsci-13-01118-s001.zip › Figure S4.pdf]

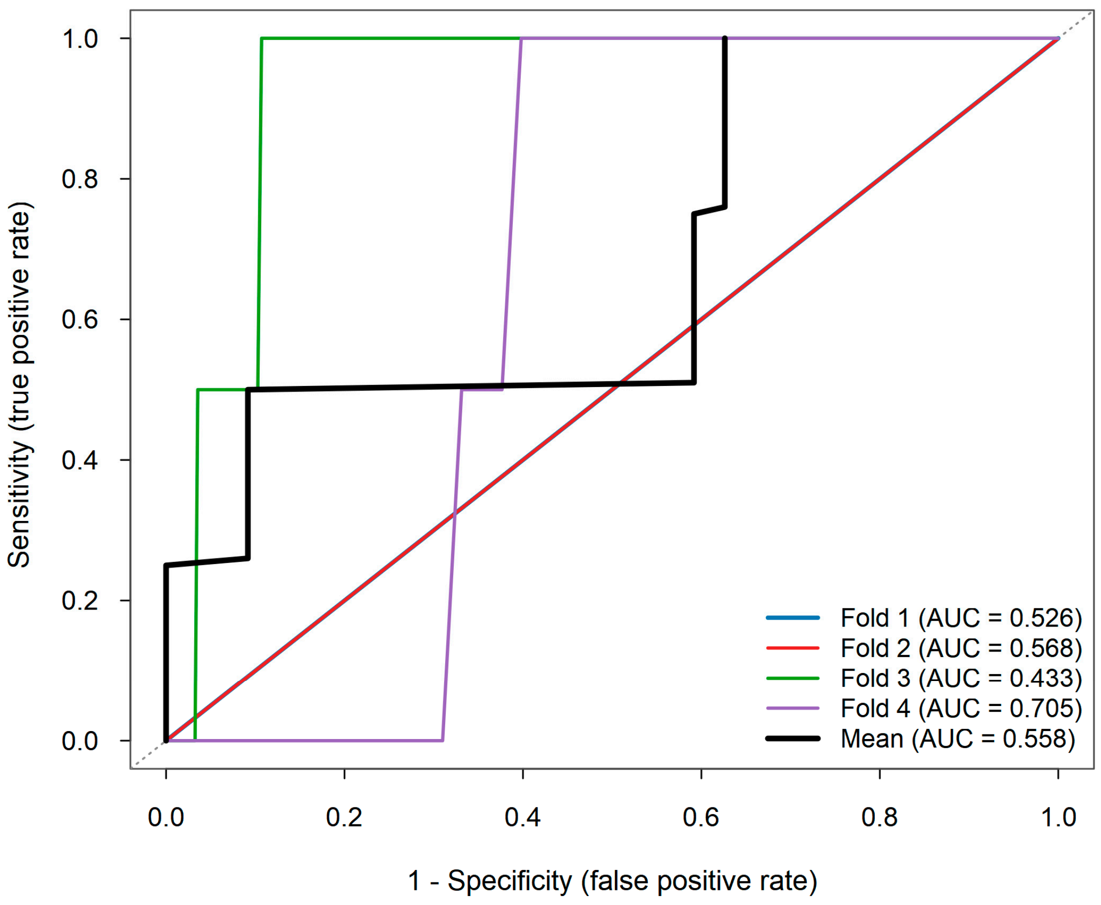

Figure S4. Receiver operating characteristic (ROC) curves of the exploratory model under four-fold spatial block cross-validation. Each panel-listed fold is plotted separately and the heavy black line is the mean curve; the mean area under the curve is 0.558 (fold values: 0.526, 0.568, 0.433 and 0.705). With two occurrence units held out per fold the curves are coarse and the AUC is unstable, which is the quantitative statement of the weak discrimination referred to in Section 3.5 and Section 4.
